# Surgical Management of Advanced Achalasia With Sigmoid Esophagus: A Case Report

**DOI:** 10.7759/cureus.21639

**Published:** 2022-01-26

**Authors:** Lu Vivian F, Dushyant S Dahiya, Connor B Shea, Faiz Tuma

**Affiliations:** 1 General Surgery, Central Michigan University College of Medicine, Saginaw, USA; 2 Internal Medicine, Central Michigan University College of Medicine, Saginaw, USA

**Keywords:** achalasia, sigmoid esophagus, dor fundoplication, heller myotomy, esophagogastroduodenoscopy

## Abstract

The surgical management of achalasia with sigmoid esophagus involves multiple significant challenges due to the difficulty in endoscopic assessment, esophageal motility disorders, and potential complication and recurrence rates. We report a 34-year-old female with worsening dysphagia and malnourishment due to advanced achalasia. An esophagogastroduodenoscopy (EGD) revealed an esophageal dilation, tortuosity, and distal blockage with undigested food. Esophagram demonstrated the typical bird beak appearance with a tortuous dilated esophagus. She underwent a laparoscopic Heller myotomy with Dor fundoplication with no complications. She was discharged on the second postoperative day, tolerating clear liquids, and then a normal diet within six weeks. Several treatment options exist for the surgical management of a sigmoid esophagus with achalasia, but there is no clear gold standard. In our case, Heller myotomy with Dor fundoplication provided favorable results, but treatment should be individualized for each case.

## Introduction

Achalasia is a neuromuscular disease of the esophagus characterized by the failure of the lower esophageal sphincter (LES) to relax, along with weak to absent esophageal peristalsis. The incidence and prevalence of achalasia are 1.63/100,000 and 10.82/100,000, respectively [1]. Increased incidence has been reported by recent studies [2]. This is likely due to improved diagnostic tests and clinical awareness. Patients with achalasia present with various degrees of symptoms, the most common of which are dysphagia for solids and liquids, occasional regurgitation of undigested food material, dyspepsia, weight loss, and chest pain [3]. Dysphagia is usually the primary and most important symptom as it occurs in over 90% of achalasia patients [3]. The type of dysphagia in achalasia is neuromuscular, which occurs with both solids and liquids, as the neuromuscular forces required to propel the bolus effect solids and liquids in a similar fashion [4]. Achalasia may often present with nonspecific gastroesophageal symptoms, which may lead to delay or confusion in the diagnosis with other common alimentary tract diseases. Hence, various investigations are often needed to identify and confirm the diagnosis [4].

Achalasia is caused by various etiological factors that are unclear at times. A multifactorial etiology has been suggested by some studies [2]. However, the diagnostic criteria of achalasia are well-established. Patients suspected of achalasia undergo and are evaluated with a combination of serial tests, including upper gastrointestinal barium study (UGI), esophagogastroduodenoscopy (EGD), and chest computerized tomography (CT) when needed [5]. Ultimately, a definitive diagnosis is made with an esophageal function test (manometry and impedance) [6].

In advanced achalasia, the esophagus gradually dilates in a progressive pattern that can be severe and resemble the shape of a sigmoid. The sigmoid esophagus is defined as an esophageal dilatation of more than 10 cm and/or tortuous shape [7]. Treatment for advanced stage achalasia with the sigmoid esophagus is challenging. Surgical and non-surgical treatments, such as mechanical pneumatic dilation, botulinum toxin injections, and myotomy, are rendered less effective in the long term, often leading to a need for re-treatment [8]. This article presents a case study of a patient with late-stage achalasia and sigmoid esophagus who was successfully treated with laparoscopic Heller myotomy and Dor fundoplication.

## Case presentation

**Figure 1:**
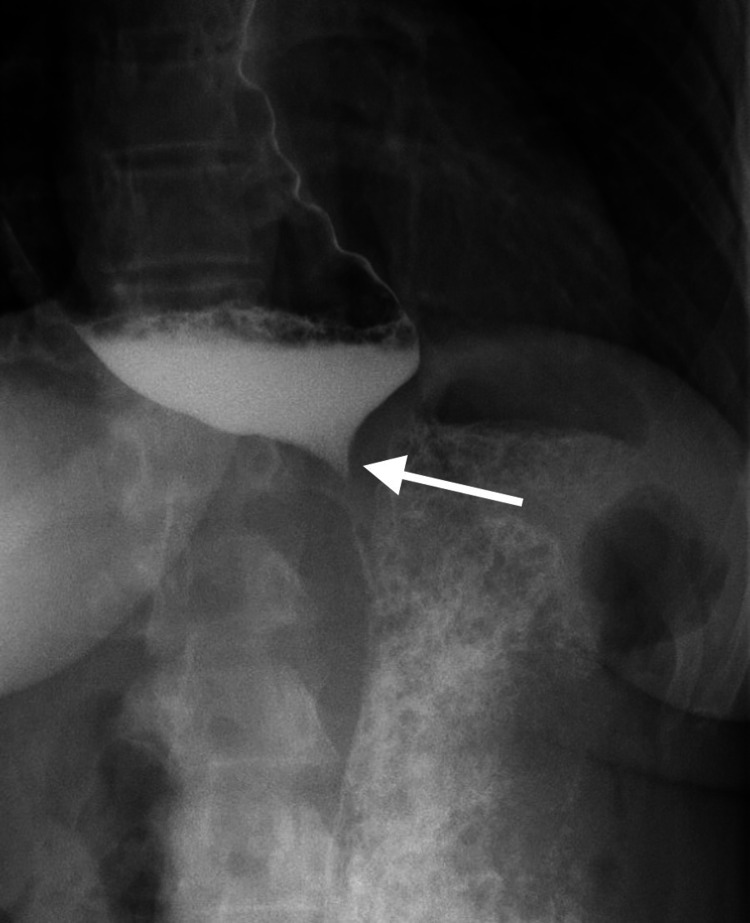
A barium swallow depicting the typical “bird’s beak” narrowing at the lower esophageal sphincter

**Figure 2:**
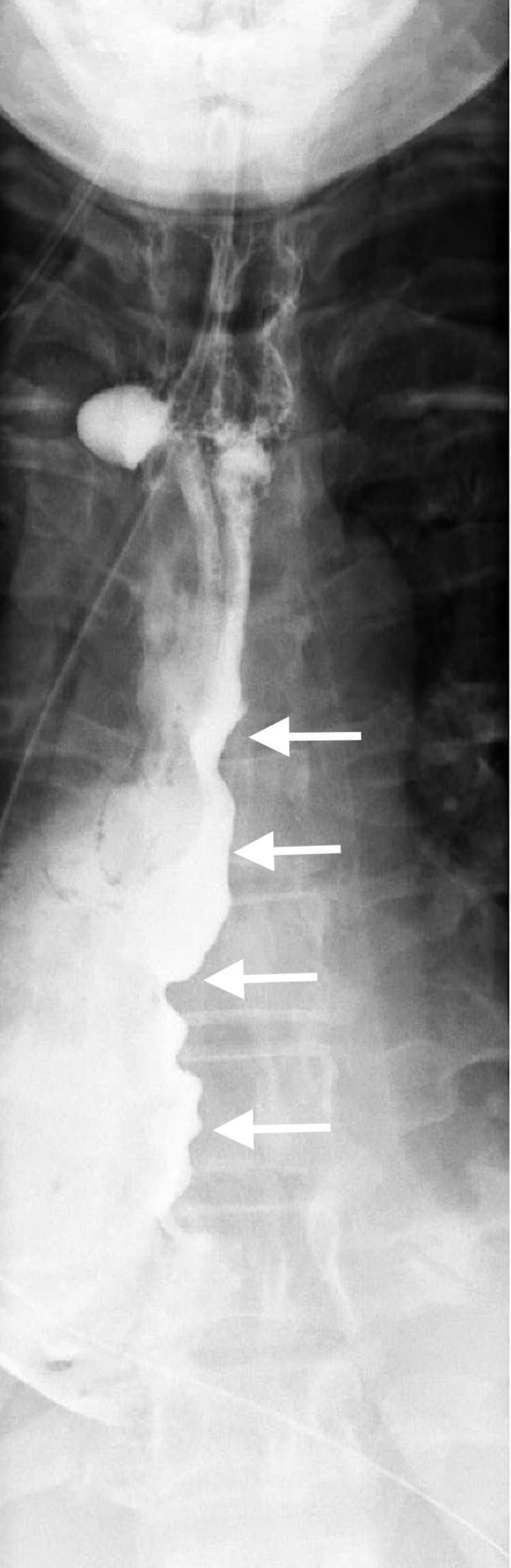
A barium swallow showing the dilated tortuous esophagus

**Figure 3:**
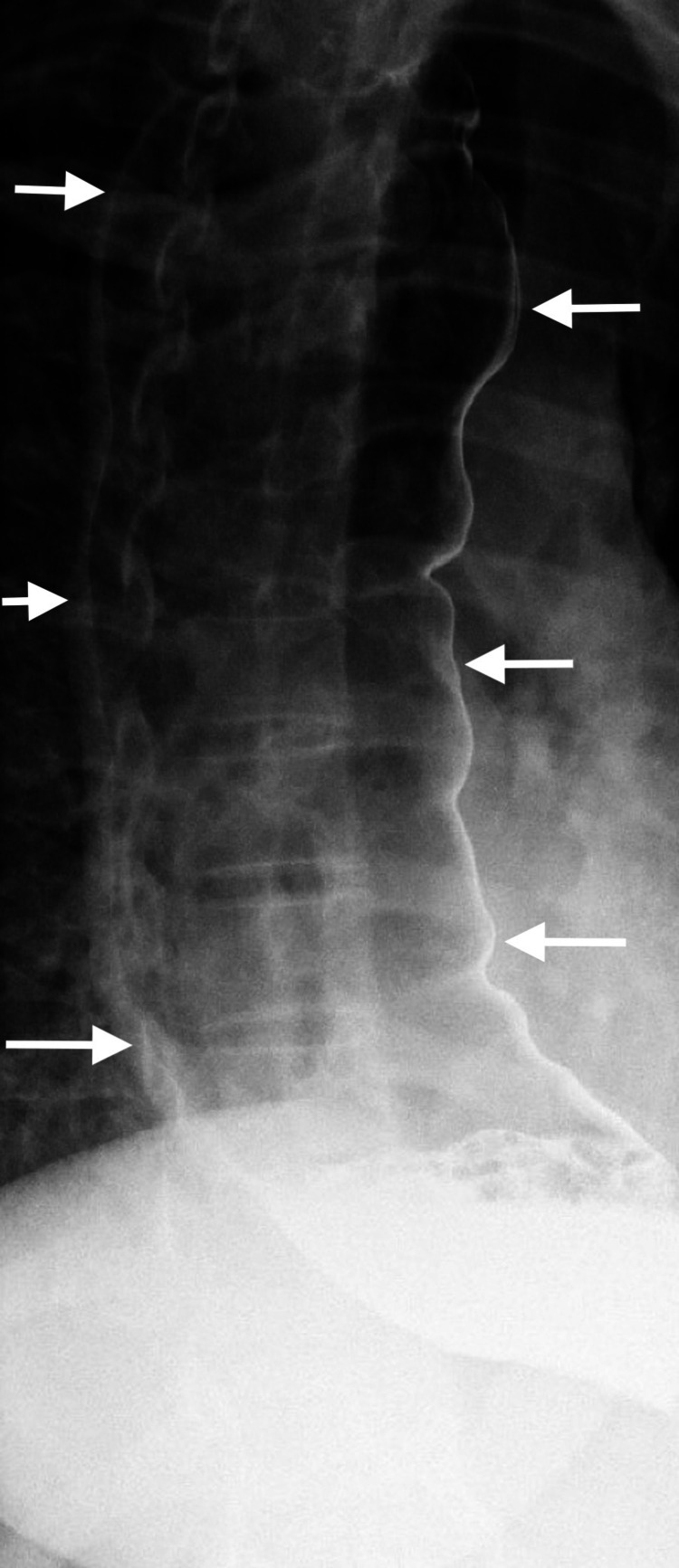
Barium swallow of the dilated sigmoid esophagus

A 34-year-old female was referred by her primary care provider to the surgical department for a two-year history of dysphagia, intermittent regurgitation of undigested food, epigastric pain, and early satiety. She had a long-standing history of gastroesophageal reflux disease (GERD) controlled with Prilosec. An EGD was attempted on two separate occasions, but both were unsuccessful due to the presence of undigested food in the lower esophagus, even after a two-day fasting period. An upper gastrointestinal (GI) barium study was then performed, which revealed a dilated thoracic esophagus with smooth tapering at the esophageal junction resembling the typical “bird beak” appearance (Figures 1-2) along with a sigmoid esophagus (Figure 3). Based on the radiological and endoscopic findings, the provisional diagnosis of achalasia was made. The patient underwent esophageal manometry but revealed poor esophageal function. It could not measure the lower esophageal sphincter resting tone. Although esophageal manometry was unlikely to change the overall treatment strategy, it was performed for confirmation, characterization of the condition, and insurance requirements. Given the patient’s continued dysphagia with significant lifestyle impairments and low surgical risk, laparoscopic Heller myotomy and Dor fundoplication were offered to the patient after reviewing the treatment options.

**Figure 4:**
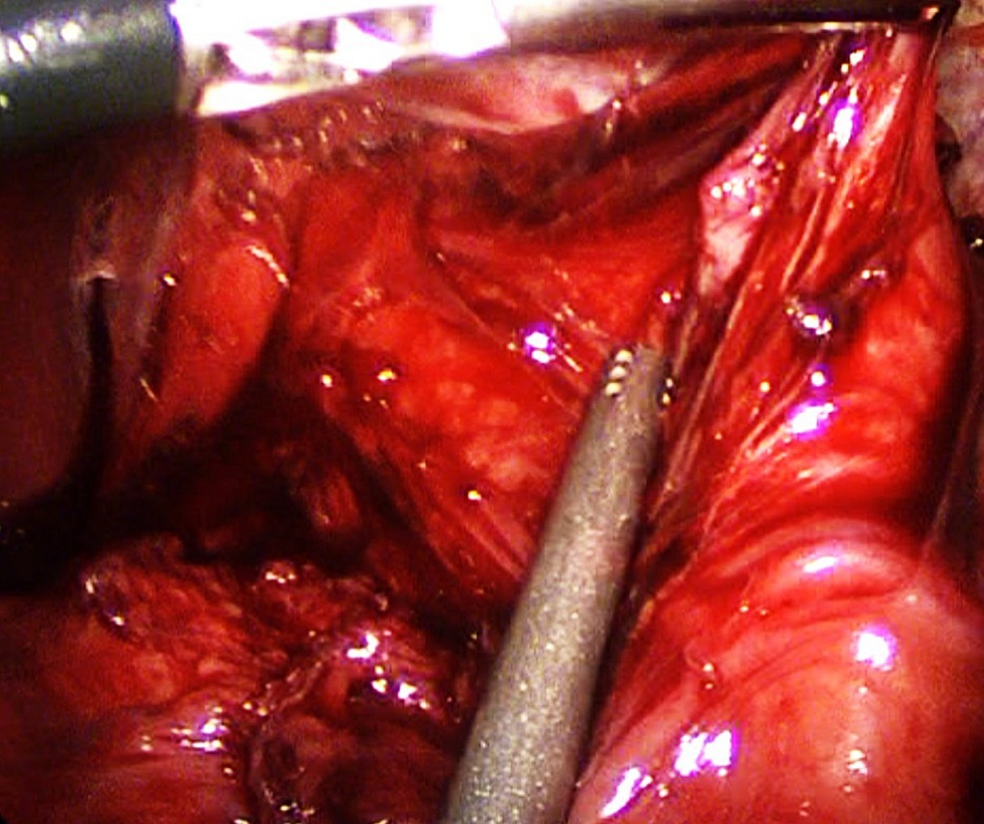
The completed 8 cm esophageal myotomy

**Figure 5:**
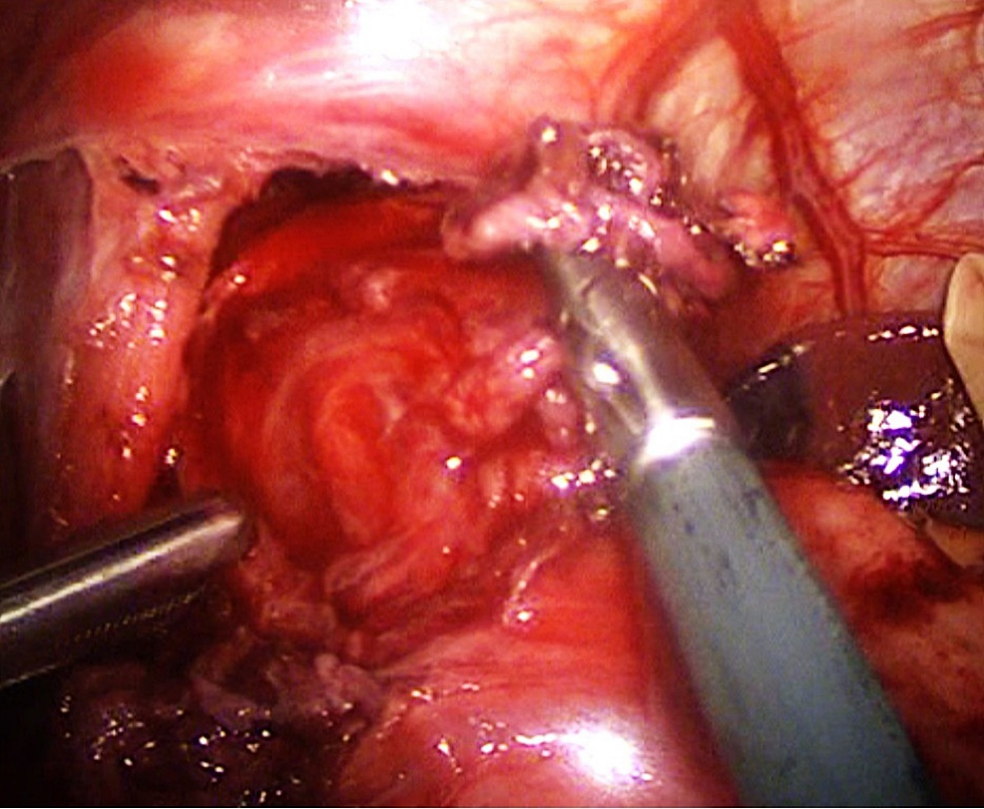
Exposed esophageal myotomy after the careful division of the longitudinal muscle fibers of the esophagus

Intraoperatively, the anterior myotomy was completed to 8 cm on the esophagus and 2.5 cm at the proximal stomach (Figures 4-5). A cruralplasty to recreate an appropriate hiatus with Dor fundoplication was performed. An intraoperative EGD was performed at the end of the procedure and revealed the same previous findings of a dilated and tortuous esophagus with food remnants. The stomach was intubated for the first time due to the straighter lower esophageal end after the mobilization. The integrity of the lower esophagus lumen and wall, the gastroesophageal junction (GEJ), and the stomach was confirmed. The procedure was completed without complications. She had an uneventful postoperative in-hospital course, continued to show improvement, and was able to tolerate clear liquids. The esophagram confirmed easier passage of the contrast to the stomach. Due to an improvement in the patients’ overall status, a decision was made to discharge her with instructions to remain on a clear liquid diet for a week and then transition to a full liquid diet. Within six weeks, she was able to tolerate a normal diet without dysphagia or regurgitation and was satisfied with her improvement.

## Discussion

Diagnosis of achalasia is unchallenging. UGI series often visualize the esophagus anatomy, including a classic “bird beak” appearance of the distal esophagus and esophageal body dilation. In advanced stage achalasia, severe esophageal body dilation results in a sigmoid-like appearance in the distal esophagus [9-10]. An EGD exam is important to visualize the mucosa and lumen to evaluate for esophagitis, reflux disease, stenosis, and malignancy but is a limited diagnostic tool, as food remnants are relatively common in achalasia [9,11]. Manometry as part of the esophageal function test is considered the gold standard test to confirm the diagnosis of achalasia. Lack of esophageal body peristalsis and absent or incomplete relaxation of the LES is the typical finding [1]. Our patient had the typical UGI series and EGD findings. However, manometry could not be completed due to patient intolerance.

The goal of achalasia treatment is to relieve symptoms and improve nutrition, which can be achieved surgically [12]. The main surgical treatment is myotomy of the distal esophagus to release stenosis with the addition of fundoplication to protect from GERD development. Esophagectomy is occasionally considered in advanced cases, however, esophagectomy is associated with significant complications such as persistent regurgitation, anastomotic leakage, and cervical fistula [13]. In our case, the decision was made to proceed with myotomy and Dor fundoplication, as it is a highly effective and safe treatment modality. The endoscopic treatment option was not preferred because of the extremely dilated and thin-walled esophagus, large food stagnation at the distal esophagus, and the necessity of achieving radical improvement. The two edges of the myotomy were widely spaced and the chance of reclosing and scarring was minimal. Also, the exposed esophageal mucosa at the base of the myotomy was fairly thin and would benefit from protection with an anterior wrap - Dor. Ultimately, our experience yielded favorable results that exceeded expectations compared to the expected prognosis.

## Conclusions

Surgical management of severe achalasia with sigmoid esophagus imposes significant challenges. There are several treatment options available, but ongoing controversy yields no clear gold standard. Management should be individualized for each case. In our case, Heller myotomy with Dor fundoplication provided satisfactory symptom improvement and an excellent treatment outcome. Further research and long-term results will be more instructive of the effectiveness of this treatment modality.
